# Characterization of Betabel Extract (*Beta vulgaris*) Encapsulated with Maltodextrin and Inulin

**DOI:** 10.3390/molecules25235498

**Published:** 2020-11-24

**Authors:** Martha A. Flores-Mancha, Martha G. Ruíz-Gutiérrez, Rogelio Sánchez-Vega, Eduardo Santellano-Estrada, América Chávez-Martínez

**Affiliations:** 1 Departamento de Tecnología de Productos de Origen Animal, Facultad de Zootecnia y Ecología, Universidad Autónoma de Chihuahua, Periférico Francisco R. Almada km 1. Chihuahua, Chih 33820, Mexico; 99azu.flores@gmail.com (M.A.F.-M.); rsanchezv@uach.mx (R.S.-V.); esantellano@uach.mx (E.S.-E.); 2 Departamento de Investigación y Posgrado, Facultad de Ciencias Químicas, Universidad Autónoma de Chihuahua, Circuito Universitario s/n Campus Universitario 2, Chihuahua, Chih 31125, Mexico; mruizg@uach.mx

**Keywords:** betalains, encapsulation, lyophilization, antioxidant activity, polyphenols

## Abstract

Betalains are powerful antioxidants contained in beets. These are divided into betacyanins (red-violet) and betaxanthins (yellow-orange), and they can be used as natural colorants in the food industry. The effects of freeze-drying pure beet juice (B) and the encapsulation of beet juice with a dextrose equivalent (DE) 10 maltodextrin (M) and agave inulin (I) as carrier agents were evaluated. The powders showed significant differences (*p* < 0.05) in all the variables analyzed: water absorption index (WAI), water solubility index (WSI), glass transition temperature (T_g_), total betalains (TB), betacyanins (BC), betaxanthins (BX), total polyphenols (TP), antioxidant activity (AA, via 2,2’-azino-bis(3-ethylbenzothiazoline-6-sulfonic acid)) (ABTS), and 2,2-diphenyl-1-picrylhydrazyl (DPPH)) and total protein concentration (TPC). The highest values of antioxidant activity were found in the non-encapsulated beet powder, followed by the powder encapsulated with maltodextrin and, to a lesser extent, the powder encapsulated with inulin. The glass transition temperature was 61.63 °C for M and 27.59 °C for I. However, for B it was less than 18.34 °C, which makes handling difficult. Encapsulation of beet extract with maltodextrin and inulin by lyophilization turned out to be an efficient method to increase solubility and diminish hygroscopicity.

## 1. Introduction

The beet (*Beta vulgaris rubra*) is a member of the Chenopodiaceae family [1]. This family includes important food crops such as chard (Beta vulgaris cicla) and spinach (Spinacia oleracea) [2]. It is a nutritious food that is generally consumed in juices, salads, pickled or cooked [3]. Beet extract has been used as a pigment in the cosmetic, pharmaceutical, and food industries [4,5,6,7,8,9,10,11,12,13]. Also, it is a potential source of nutrients and can be used as an ingredient in making healthy foods. [14]. Beets contain betalains, a unique class of antioxidants [15,16]. These are classified into two groups: betacyanins, which exhibit a red-violet coloration, and betaxanthins, which impart a yellow-orange pigmentation [4,17,18,19,20]. Beet extract is a color approved under code 73.40 by the FDA [21] and by the EU designated with the number E162 [1,22].

Betalains have low stability to various factors, such as high temperatures [23], alkaline pH [24], enzymatic activity [25], presence or absence of light [26], oxygen and/or metals [18,27]. Due to the instability of betahalamic compounds, their use has been restricted in food [19,28,29,30,31,32,33]. Encapsulation has been used to protect bioactive compounds contained in foods such as essential oils, vitamins, antioxidants, bactericides, colorants, flavorings, among others [34,35,36,37]. This is a process by which phytochemicals are coated with a thin wall of a protective material called a carrier or encapsulating agent [38,39,40,41,42,43]. Encapsulation of betalains in different edible matrices has been shown to increase their stability and maintain its antioxidant activity [3,28,44,45,46,47,48,49,50]. There are a great variety of techniques to carry out encapsulation, some authors have classified them as chemical and physical [51]. At the time of encapsulation, it should be taken into account that there are complications related to caking, particle agglomeration and stickiness, as a result of the low glass transition temperature (T_g_) [52]. Due to these, it is necessary to use encapsulating agents that increase said parameter (high molecular weight and low viscosity) [43]. Among the physical or mechanical techniques appear: fluidized bed drying, extrusion, lyophilization and spray drying [53,54]. However, little has been studied on the properties of the bioactive compounds found in beet extract encapsulated by lyophilization. Therefore, the physicochemical characteristics of these powders could be evaluated due to their use as a pigments with antioxidant activity; since they represent a good alternative for the development of new functional food products. The objective of this study was to evaluate the effect of encapsulation by lyophilization on the physicochemical characteristics (water absorption index, water solubility index and glass transition temperature) and bioactive compounds (total betalains, betacyanins, betaxanthins, total polyphenols, antioxidant activity and total protein concentration) of betabel extract.

## 2. Results and Discussion

**Figure 1:**
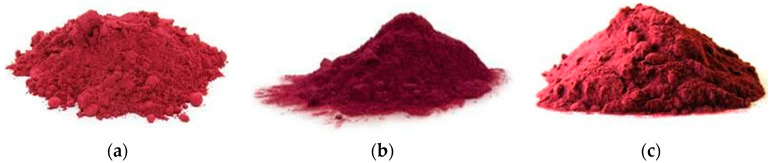
Freeze-dried beet powders: (**a**) pure beet powder; (**b**) maltodextrin encapsulated beet powder; (**c**) beet powder encapsulated with inulin.

The powders (Figure 1) presented significant differences (*p* < 0.05) in all variables analyzed: water absorption index (WAI), water solubility index (WSI), glass transition temperature (T_g_), total betalains (TB), betacyanins (BC), betaxanthins (BX), total polyphenols (TP), antioxidant activity (AA) (via 2,2’-azino-bis(3-ethylbenzothiazoline-6-sulfonic acid)) (ABTS), and 2,2-diphenyl-1-picrylhydrazyl (DPPH)) and total protein concentration (TPC). Table 1 shows the physicochemical characterization of beet juice powders.

### 2.1. Yield of Encapsulation

The yield of the powders was found in a range from 35,772 to 65,329 g/100 g, with the highest percentage presented in the powder encapsulated with dextrose equivalent (DE) 10 maltodextrin (M) (65,329 g/100 g), while the powder encapsulated with agave inulin (I) indicated 61,678 g/100 g. The non-encapsulated powder (B) had a yield of 35,772 g/100 g, which was the lowest yield. The yield values found in this study are greater than the values found by Castro-Muñoz Saenz et al., 2009 [55] in the encapsulation of clarified juice from purple cactus pear (*Opuntia stricta*), using a combination of maltodextrin and gelatin (range yields between 7.76–14.85 g/100 g). Meanwhile, Obón et al. (2009) [56] reported yield values of 58 g/100 g using glucose syrup as an encapsulant agent. Likewise, Saenz et al., 2009 [57] mentioned yield values between 23 and 81 g/100 g using maltodextrin or inulin. All these authors used spray drying for the encapsulation. The high yield values found in this study may be due to the affinity of the wall material, the encapsulated compounds [47,48], and the higher recovery and lower degradation of betalains during freeze-drying compare to spray drying [48,49].

### 2.2. Water Absorption Index (WAI)

In the WAI, percentages of 44.01 ± 1.000, 27.09 ± 6.096 and 30.68 ± 3.427 were obtained for B, M and I, respectively. No significant differences were found for I and M values. Higher WAI observed on B may be due to the fact that the hydrophilic functional groups of polyphenols, betalains, betacyanins and betaxanthins are free and can form hydrogen bonds with water molecules. Betacyanins pigments quickly absorb humidity from the environment due its hydrophilic groups and the encapsulation with maltodextrin protects the pigment by allowing the drying process and also helps to reduce hygroscopicity by increasing their stability [58]. Moreover, it would have been expected that M would show higher WAI values because maltodextrin is more soluble in water than inulin [59]. However, the values could be due to the fact that maltodextrin occupied its binding sites (hydrophilic functional groups) with polyphenols, betalains, betacyanins and betaxanthins, leaving fewer binding sites available to bind with water. The WAI results found in this work are higher than those reported by other authors [60] who indicated 6.04% of WAI in prickly pear powder encapsulated with soluble barley fiber. Likewise, lower results than those of this research have been reported for the WAI in barley flour (6.42 and 7.26%), barley-tomato flour (6.10 to 7.03%) and barley-grape flour (7.85 % to 15.79%). However, the powders in this study were obtained by extrusion [61]. Also, WAI values of 1.92, 4.48 and 2.31% were reported in wheat, potato and quinoa flours, respectively [62]. The WAI is related to the humidity of the product under certain conditions of relative humidity and temperature; and it can be used as an indication of the degree of modification of starches by thermomechanical treatments [62].

### 2.3. Water Solubility Index (WSI)

**Figure 2:**
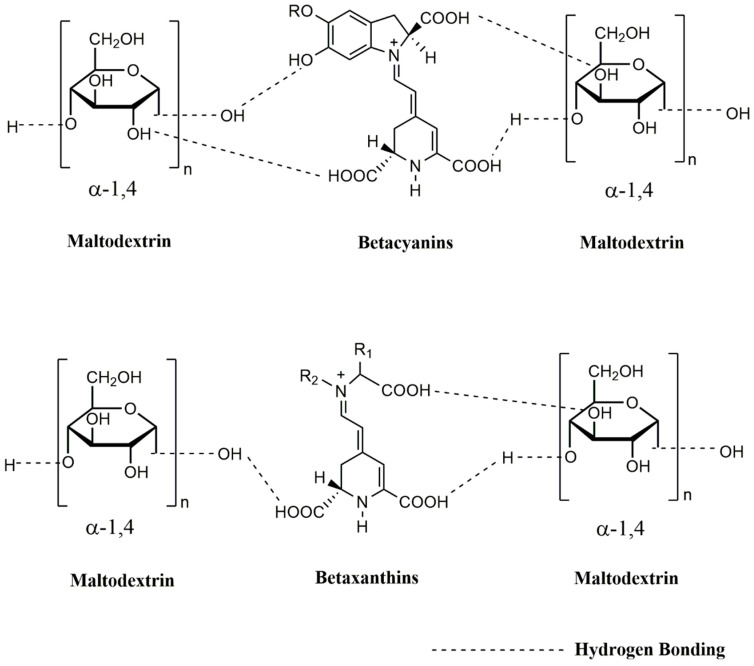
Interaction between maltodextrin with betaxanthins and betacyanins.

The WSI values were 8.95 ± 0.023%, 9.05 ± 0.016% and 9.37 ± 0.014% for B, I and M, respectively. This indicates that when exposed to water, the powder encapsulated with M presents greater solubility (*p* < 0.05) compared to B and I. Water holding capacity is usually defined as the amount of water retained or absorbed by a specific compound after acquiring the state of equilibrium. High water holding capacity is directly related with the presence of hydrophilic regions in the chemical structure [63]. This is also associated with dextrinization and used as an indicator of the degradation of the molecular structure of polymers present in starch and dietary fiber [64]. I presented the lowest value of WSI, this could be due to the decrease of the hydrophilic groups available to bind water, that could be occupying linking polyphenols, betalains, betacyanins and betaxanthins (Figure 2). Besides, M presented the highest values of WSI, this could be due to the fact that maltodextrin has a high capacity to absorb water of 72–83% [55] compare to inulin 15–49% [65]. The results found in this work are higher than those reported [66] in prickly pear powder encapsulated with soluble barley fiber, made by spray drying, where values of 0.24 and 0.76% were reported for the WSI. Likewise, the WSI was evaluated in wheat, potato and quinoa flours, and reported percentages of 2.09, 7.45 and 5.10 [62], respectively. On the other hand, the WSI values obtained in this study are within the range reported in barley flour (6.27 to 9.67%), barley-tomato flour (7.08 to 12.99%) and barley-grape flour (7.85 to 15.79%). Nevertheless, the powders in these study were obtained by extrusion [61]. Advantages of using polysaccharides to encapsulate betalains include their solubility, bland flavor, low hygroscopicity and ability to protect bioactive compounds from oxidation.

### 2.4. Glass Transition Temperature (T_g_)

The T_g_ values presented had significant differences (*p* < 0.05) between the powders, with values of 18.34 °C for B, 27.59 °C for I, and 61.63 °C for M. This property is important since it is related to the stability of the product [66]. Low T_g_ values indicate a high hygroscopicity of the powder [67], with B being the powder with the greatest capacity to absorb moisture. This result is due to the fact that the hydrophilic groups of betalains, betacyanins, and betaxanthins are available to form hydrogen bonding with water. While in encapsulated products, these binding sites are used to form hydrogen bonds with the hydrophilic functional groups of maltodextrin and inulin (Figure 2). Betacyanin pigments quickly absorb humidity from the environment due to their hydrophilic groups, and the encapsulation with maltodextrin protects the pigment by allowing the drying process and when at the same time, helps to reduce hygroscopicity and increase their stability [58]. In general, the T_g_ values obtained in this investigation were lower than those reported in other studies, in which the T_g_ was in the range of 32.27 to 387.10 °C [3,28,47,66,67]. The addition of encapsulating agents such as maltodextrins, inulins, and gums have been shown to lead to a considerable increase in T_g_, largely confirming the stability of betalains [3,28,47,66,67]. Also, it has been reported that beet juice encapsulated with Arabic gum has T_g_ values between 98.96 and 105.86 °C when using the spray drying method [3]. Likewise, the encapsulation of beet pigments with maltodextrin and acacia has a T_g_ of 90 °C [47]. Meanwhile, red prickly pear betalains encapsulated with soluble barley fiber by spray drying obtained a T_g_ in the range of 32.27 to 38.11 °C [66]. In amaranth, betalains encapsulated by means of spray drying with maltodextrin, a T_g_ of 54.7 °C was reported [67].

### 2.5. Total Betalains Content (TB)

**Figure 3:**
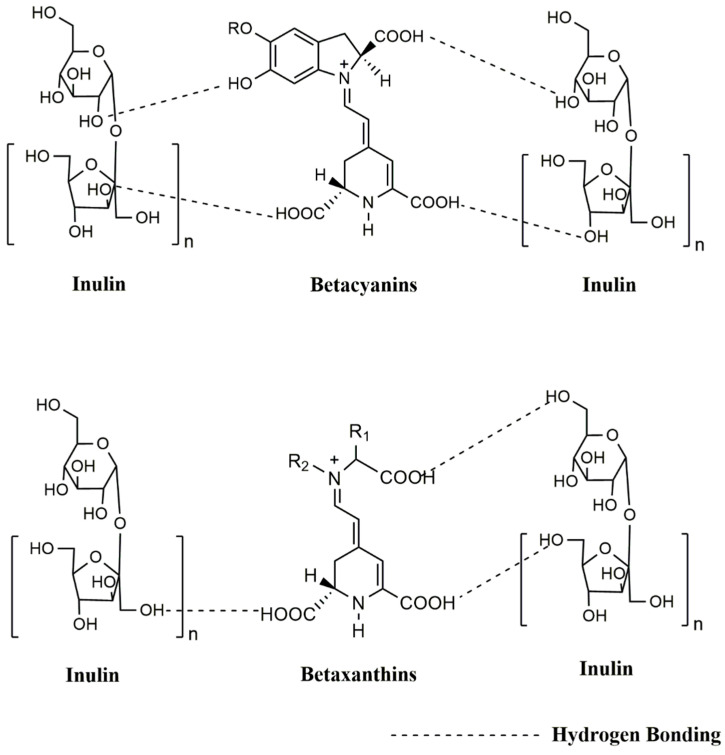
Interaction between inulin with betaxanthins and betacyanins.

The average betalain content of beets is approximately 130 mg/100 g [68,69]; however, new varieties of beets produce around 450–500 mg/100 g of betahalamic pigments [70]. Table 1 shows the TB content of this study. The concentration of TB in B was 382.35 ± 0.092, being 219.17 ± 0.092 mg/100 g of BC and 163.17 ± 0.001 mg/100 g of BX. The content of encapsulated TB (M = 15.71 ± 0.016 mg/100 g; I = 10.11 ± 0.016 mg/100 g) in this investigation did not coincide with the results obtained by other authors who reported a TB concentration lower than 121.6 mg/100 g [49] in unencapsulated beet powder. Other studies have reported 77.70 mg/100 g, 28.82 mg/100 g and 11.97 mg/100 g of TB in beet powders [45,47,71]. However, these authors used maltodextrin and Arabic gum and mixtures of both agents to encapsulate the powder by spray drying. On the other hand, the TB content in prickly pear powders encapsulated with maltodextrin and gelatin in different proportions ranged from 11.33 to 35.93 mg/100 g [55]. Furthermore, the TB content of 49.1 to 52.1 mg/100 g was reported in powders of prickly pear encapsulated with K-4484 and Capsul^®^ by spray drying [72]. Also, values between 42.0 to 88.0 mg/100 g of TB have been reported in powders of prickly pear encapsulated with maltodextrin and inulin by spray drying [57]. The higher stability and, as a consequence, the presence of betacyanins over betaxanthins may be due to the fact that some of them have a glycosylated structure, which has a high oxidation-reduction potential [30,31,71]. Variation in anthocyanin content is related to the type of encapsulated agent and its behavior during freeze-drying [73]. The encapsulation efficiency depends on the capacity of the wall materials to hold in the core of the microparticles the component to encapsulate; in this case, it is attributed to the capability of the biopolymeric wall materials (maltodextrin and inulin) to interact with betalains. Maltodextrin and inulin alone can interact with betacyanins and betaxanthins through hydrogen bonding (Figure 2 and Figure 3).

### 2.6. Total Polyphenols Content (TP)

The content of TP (Table 1) was found in a range of 5.97 to 12.35 milligrams gallic acid equivalent per gram (mg GAE/g) in this study. For B it was 12.35 ± 0.001 mg GAE/g, being the powder with the highest content of total polyphenols, followed by M with a value of 6.09 ± 0.001 mg GAE/g and then I where 5.97 ± 0.001 mg GAE/g was found. These results are higher than those previously reported by other authors, who reported 4.88 mg GAE/g TP in maltodextrin encapsulated beet powder by lyophilization [49]. In prickly pear powder encapsulated with soluble fiber by spray drying, ranges from 14.2 to 16.5 mg GAE/g of TP content were reported [66]. Also, values of 1.81 to 2.41 mg GAE/g have been mentioned in terms of TP content in prickly pear powders encapsulated with maltodextrin and inulin by spray drying [57]. On the other hand, TP content between 0.41 and 0.81 mg GAE/g has been reported in yellow pitahaya powder encapsulated with maltodextrin by spray drying [43].

### 2.7. Antioxidant Activity (AA)

The AA of the beet powders (B, M, and I) was maintained in a range of 0.65 to 0.91 mM TE/100 g using the ABTS methodology and DPPH from 0.21 ± 0.001 to 0.45 ± 0.001 mM TE/100 g, with significant differences (*p* < 0.05) between the powders using both techniques. The highest AA was for the beet powder without carrier agent (B) with an ABTS value of 0.90 ± 0.001 mM TE/100 g and 0.44 ± 0.001 mM TE/100 g using DPPH. The beet extract encapsulated with maltodextrin (M) was ranked second with values of 0.74 ± 0.001 mM TE/100 g in the ABTS technique and using DPPH with 0.29 ± 0.001 mM TE/100 g, while the extract encapsulated with inulin (I) presented the lowest antioxidant activity with a value of 0.64 ± 0.001 mM TE/100 g and 0.20 ± 0.001 mM TE/100 g, with ABTS and DPPH, respectively. The values obtained in this research are higher than those previously reported in yellow pitaya powders encapsulated with maltodextrin by spray drying (0.07 mM TE/100 g for ABTS and 0.09 mM TE/100 g using DPPH) [43]. In prickly pear powders encapsulated with maltodextrin and inulin by spray drying, AA values ranged from 0.004 to 0.029 mM TE/100 g using the DPPH method [55].

### 2.8. Total Protein Concentration (TPC)

Significant differences (*p* < 0.05) were found in the TPC. The values were 5.974 ± 0.001 for beet extract without encapsulation (B), 3.524 ± 0.001 for the beet extract encapsulated with maltodextrin (M), and 3.655 ± 0.001 for the beet extract encapsulated with inulin (I).

### 2.9. Correlation between Variables

Table 2 shows the correlation coefficient (r) and its level of significance between the response variables of the lyophilized beet extracts (B, M, and I). All powders showed similar behaviors in the variables analyzed: total betalains (TB), betacyanins (BC), betaxanthins (BX), total polyphenols (TP), antioxidant activity (AA) by both methods (ABTS and DPPH), and total protein concentration (TPC).

Among the most important correlations, a positive correlation was found between AA and TP (r = 0.9398 in ABTS, and r = 0.9388 in DPPH *p* < 0.0002). This may be because polyphenols are powerful antioxidants [74]. A positive correlation was found between AA and TB (r = 0.9293 in ABTS and r = 0.9283 in DPPH, *p* < 0.0003). This could be due to the presence of bethalamic acid since it acts as an antioxidant agent. Similar results have been reported in red prickly pear extract [55]. A positive correlation was found between AA and TPC (r = 0.9500, in ABTS and r = 0.9491 in DPPH, *p* < 0.0001). This could be attributed to the fact that sugars and proteins generate products with high antioxidant activity [75], which can be observed despite the presence of betalains [55]. A positive correlation between TB and TP was also found (r = 0.9996, *p* < 0.0001). This could be because betalains are the main polyphenols present in beets [49]. A negative correlation was found between WAI and T_g_ (r = 0.7410, *p* < 0.0224), which may be due to the powders beginning to hydrate at relatively low temperatures [45].

## 3. Materials and Methods

### 3.1. Materials

The beet was acquired in a local market in Chihuahua, Mexico, during the period from March to July 2018. In addition, maltodextrin (DE 10) and agave inulin, both from Sigma-Aldrich^®^, St. Louis, MO, USA, were used as carrier agents in the encapsulation of beet juice. The powders were characterized in terms of water absorption and solubility index, glass transition temperature, total betalains content, betacyanins, betaxanthins, total polyphenols content, antioxidant activity by the ABTS and DPPH methods, and total protein concentrations.

### 3.2. Reagents

The 2,2’-azino-bis(3-ethylbenzothiazoline-6-sulfonic acid) (ABTS), 2,2-diphenyl-1-picrylhydrazyl (DPPH), Trolox, ammonium salt, potassium persulfate, Folin-Ciocalteu, sodium carbonate, gallic acid, trichloroacetic acid, citrate, and phosphate standard reagent were purchased from Sigma-Aldrich (St. Louis, MO, USA). High-performance liquid chromatography (HPLC)-grade methanol and HPLC-grade water were purchased from J. T. Baker (Mexico City, Mexico). Deionized water was obtained using a deionizer (Barnstead, Thermo Scientific, Waltham, MA, USA). All other reagents used were analytical grade.

### 3.3. Preparation of Beet Juice Powders

#### 3.3.1. Obtaining Beet Juice, Encapsulation, and Lyophilization

The beet juice was extracted with a domestic extractor (Cold Press 900W, Breville, Syndney, Australia). The encapsulating agents (Maltodextrin-M and Inulin-I) were added following the technique used by Antigo et al., 2017 [49] in an amount of 30 g of dry matter per 100 mL to the red beet juice at room temperature. Then mixtures were homogenized for 10 min (Vortex-Ultra-Turrax IKA T18 basic) with a dispersion tool (S18N-19G, IKA Works Inc., Wilmington, NC, USA). For encapsulation by freeze-drying, the samples were frozen for 48 h at −20 °C. Then, these were dried by freeze-drying for four days (at −85 °C and 0.035 mbar pressure) to ensure complete drying of the product (Freeze-dryer Labconco Niro Mobile Minor DK-2860, GEA Company, Kansas City, MO, USA). Three powders were obtained: pure beet (B), beet extract encapsulated with maltodextrin (M), and beet extract encapsulated with inulin (I). Powders were stored at 9 ± 3 °C for later analysis.

#### 3.3.2. Yield of Encapsulation

The lyophilization yield was calculated using the technique described by Castro-Muñoz et al., 2015 [55], by determining the recovered powder; according to the following equation

(1) YE (%)=[(W2/W1)×(100)]

where YE = the yield (g 100 g^−1^), W_2_ = weight (g) of the collected product, and W_1_ = weight (g) of mass in the feed.

#### 3.3.3. Aqueous Extract

The extracts used to determine the content of total polyphenols and antioxidant activity were obtained according to Pitalua et al., 2010 [45] with some modifications. Briefly, 0.5 g of powder was dispersed in 10 mL of methanol and deionized water (1:1 *v*/*v*). The dispersions were homogenized for 15 s (Vortex-Ultra-Turrax IKA T18 basic) with a dispersion tool (S18N-19G, IKA Works Inc., Wilmington, NC, USA). The mixture was then centrifuged at 3000× *g* for 10 min (Centrifuge-Centra Avanti^®^ J-26 XPI. Beckman Coluter^®^, Indianapolis, IN, USA). Supernatants were filtered using a 0.42 µm pore polyethylene filter (Millipore Corp., Bedford, MA, USA) and stored frozen (−20 ± 3 °C) until analysis.

### 3.4. Characterization of Beet Juice Powders

#### 3.4.1. Water Absorption and Solubility Index

The WAI and WSI were determined according to a method previously reported by Anderson et al., 1970 [76] with some modifications. A sample of 0.5 g (Analytical Balance-PA214C, OHAUS Pioneer, Parsippany, NJ, USA) was weighed and added to 6 mL of deionized water at 30 °C and incubated (Recirculation Water Bath-WBC22, WiseCircu, Wertheim, Germany) for 30 min. Subsequently, it was centrifuged at 3000× *g* for 10 min (Centrifuge-Centra Avanti J-26 XPI, Beckman Coulter, Indianapolis, IN, USA). The supernatant was decanted into a capsule at a constant weight; the new weight was recorded (capsule + supernatant). Then it was dried at 85 °C for 24 h and subsequently weighed again (Analytical Balance-PA214C, OHAUS Pioneer, Pine Brook, NJ, USA). The WAI was calculated as the weight of the sediment obtained after removal of the supernatant per unit weight of the original solids on a dry basis. And WSI was the percentage of dry matter recovered after evaporation of the supernatant obtained from WAI. Each determination was carried out in triplicate, and an average value was obtained for each sample.

#### 3.4.2. Glass Transition Temperature

The T_g_ was determined following the method described by Ahmed et al., 2010 [77] by differential scanning calorimetry (DSC) in Thermal Analysis Instrument equipment (DSC Q-2000, Crawley, UK). A sample of 0.6 mg was placed in each 40 µL aluminum cell and sealed. Subsequently, they were subjected to different heating and cooling cycles, all at a heating ramp of 15 °C/min. The first cycle was from room temperature to 0 °C, the second cycle from 0 °C to 120 °C, the third cycle from 120 °C to 0 °C, and the fourth cycle from 0 °C to 120 °C. An empty cell was used as a reference for all measurements. As a result, thermograms were obtained in which a graph of heat flow (Y-axis) versus temperature (X-axis) was observed. These were analyzed with the Universal Analysis software to obtain the T_g_ generated in the fourth heating cycle. Each determination was made in duplicate, and an average value was obtained for each sample.

#### 3.4.3. Extraction of Betalains

The betalains extraction was carried out according to Güneşer, 2016 [12]. Aliquots of 4 mL of the aqueous extract were placed in Corning tubes and mixed with 4 mL of the trichloroacetic acid (TCA) solution at a 4% concentration. Then it was homogenized using a vortex (Ultra-Turrax IKA T18 basic) for 3 min and centrifuged at 4032× *g* (Avanti^®^ J-26 XPI. Beckman Coluter^®^, Indianapolis, IN, USA) for 10 min at 25 °C. The supernatant was filtered through a 0.45 µm pore polyethylene filter (Millipore Corp., Bedford, MA, USA). Samples were kept at −20 °C until analysis.

#### 3.4.4. Total Betalains Content

The photometric quantification of total betalains was determined following the method described by Ruíz-Gutiérrez et al., 2014 [66]. The aqueous extracts of B were diluted with McIlvaine buffer (pH 6.5, citrate-phosphate in a 1 to 10 ratio. However, for M and I, to obtain values at their respective absorption maxima, this dilution was not necessary. TB was calculated as follows

(2) B [mg/g]=[(A×DF×MW×V)/(Є×L)]

where: A = value at maximum absorption (534 for BC and 480 for BX) at 600 nm, DF = dilution factor, MW = molecular weight (550 g/mol for BC and 308 g/mol for BX), V = volume of the solution (1000 mL), Є = molar extinction coefficient (60,000 L/mol cm for BC and 48,000 L/mol cm for BX), and L = length of the reading cell (1 cm).

The quantification of BC and BX were calculated separately, and these two results were added to determine the BT content. These measurements were carried out in triplicate, and the results obtained were expressed in mg/100 g of powder.

#### 3.4.5. Total Polyphenols Content

The standard curve for TP was performed according to what was reported by Xu and Chang, 2007 [78]. For the calibration curve, a stock solution was prepared by dissolving 0.5 g of gallic acid in 250 mL of distilled water. The concentrations used were 400, 300, 200, 150, 100, 80, 60, 40, and 20 ppm.

The TP was determined following the Folin-Ciocalteu spectrophotometric method reported by Singleton and Rossi, 1965 [79] with some modifications and using gallic acid (GA) as a standard. A mixture was prepared by combining 50 µL of sample extract with 3 mL of distilled water, 250 µL of Folin-Ciocalteu reagent, and 750 µL of sodium carbonate solution (7%). The mixtures were stirred for 10 s and stood for 8 min at room temperature. Subsequently, 950 µL of distilled water was added. The mixtures stood for 2 h at room temperature in the dark. The absorbance was measured at 765 nm in a UV spectrophotometer (UV-1800. Shimadzu, Japan). Measurements were made in triplicate. The absorbance results were linearized in the regression equation (y = 0.0929x − 0.0197; r² = 0.9991) obtained from the calibration curve and were expressed in mg gallic acid equivalent (mg GAE/g).

#### 3.4.6. Antioxidant Activity

The standard curve for ABTS and DPPH was performed according to what was reported by Thaipong et al., 2006 [80]. A stock solution was prepared by dissolving 31.3 mg of Trolox (6-hydroxy-2,5,7,8-tetramethylchroman-2-carboxylic acid) in 10 mL of methanol (r^2^ = 0.99). The dilutions used were 1.20, 1.00, 0.80, 0.60, 0.40, 0.20, 0.10, 0.08, 0.05, and 0.03 mM.

- AA by ABTS methodology
  It was carried out as established by Thaipong et al., 2006 [80]. A solution of ABTS (2,2’-azino-bis (3-ethylbenzothiazoline-6-sulfonic acid)) 7.4 mM was prepared, dissolving 38.8 mg of crystallized ammonium salt of ABTS in 10 mL of distilled water. Then, a potassium persulfate solution 2.6 mM was prepared by dissolving 6.6 mg in 10 mL with distilled water. To generate the ABTS radical, these two solutions were mixed and allowed to stand in the dark at room temperature for 12 h. For the ABTS working solution, 1 mL of the ABTS free radical solution was mixed with 60 mL of methanol to reach an absorbance of 1.1 + 0.02. Subsequently, 150 µL of a sample (or standard: Trolox) and 2850 µL of ABTS working solution were placed in a 3 mL plastic cell and allowed to stand for 2 h in the dark at room temperature, then the absorbance was read at 734 nm in a UV spectrophotometer (UV-1800. Shimadzu, Japan). Measurements were made in triplicate. The antioxidant capacity was reported as equivalent mM Trolox (mM TE/100 g). For this, the absorbance obtained was substituted in the regression equation (y = −1.0726x + 0.9863; r² = 0.9967) obtained from the Trolox calibration curve.
- AA by DPPH methodology
  The antioxidant capacity was determined by the DPPH (2,2-diphenyl-1-picrylhydrazyl) method established by Thaipong et al., 2006 [80] with slight modifications. First, a stock solution of DPPH was prepared by dissolving 0.0240 g of DPPH in 100 mL of methanol to obtain a concentration of 0.6 mM. The solution was stored in an amber bottle and frozen at −20 °C until used. From this solution, the working solution was prepared, for which 10 mL of the stock solution were taken and mixed with 45 mL of methanol to obtain a final concentration of 0.1 mM and an absorbance of 1.1 + 0.02. Subsequently, 150 µL of sample (or standard: Trolox) and 2850 µL of the DPPH working solution was placed in a 3 mL quartz cell. It was allowed to stand for 3 h in the dark at room temperature, and then the absorbance was read at 515 nm on a UV spectrophotometer (UV-1800. Shimadzu, Japan). Measurements were made in triplicate. The antioxidant capacity was reported as equivalent mM Trolox (mM TE/100 g) using the absorbance obtained and substituting in the regression equation (y = −1.3055x + 1.1077; r² = 0.9994) obtained from the Trolox calibration curve.

#### 3.4.7. Total Protein Concentration

The calibration curve corresponding to the TPC was performed according to the method described by Bradford 1976 [81]. Seven standards of bovine serum albumin (BSA) were prepared at different concentrations, and the regression equation was obtained. The absorbance results were linearized in the regression equation of the calibration curve, and the peptide concentration of each of the treatments was analyzed. The dilutions used were 1.20, 1.00, 0.80, 0.60, 0.40, 0.20, and 0.10 mM.

The TPC was determined according to the method described by Bradford, 1976 [81]. The proteins react with the bright blue dye, and a product that absorbs strongly at 595 nm is obtained. For the TPC, 0.1 mL of the sample and 1 mL of the Bradford reagent were taken and allowed to react in the dark for 45 min. Once this time had elapsed, the absorbance was taken at 595 nm in a spectrophotometer (UV-1800 Shimadzu). The absorbance results were linearized in the regression equation (y = 0.1526x − 0.0597; r² = 0.9973) of the calibration curve and the total protein concentration was expressed in µg/mL of BSA.

#### 3.4.8. Statistical Analysis

Powders characterization results (measured only once in time) were analyzed through an analysis of variance (ANOVA) procedure through the following model

(3) Yij=µ+Ti+εij

where: Yij = response variable measured in the j-th repetition of the i-th treatment, µ = general mean common to all observations, Ti = effect of the i-th treatment, and εij = random error measured in the j-th repetition of the i-th treatment; which was assumed to be identically and independently distributed in a normal way with mean µ and variance σ2. When the treatments showed differences, a multiple comparison of means was performed using the Tukey’s and Duncan’s tests. The program used was SAS 9.0 (Institute Inc., Cary, NC, USA, 2006).

## 4. Conclusions

The highest values for all the variables were found in the unencapsulated beet powder (B); however, as this has a high hygroscopicity, its handling is difficult. The advantages of using maltodextrin and inulin as encapsulating agents are the high solubility, mild taste, and low hygroscopicity of the obtained powders. The powder encapsulated with maltodextrin (M) presented higher values of the bioactivities analyzed, and also, the powder had a higher T_g_, which indicates good stability.

Despite the fact that the total betalain values in the encapsulated powders were lower than those reported in other studies, the encapsulation process by lyophilization turned out to be efficient, suggesting that encapsulated beet powders are promising natural pigments that can be used in a variety of products. In addition, due to its antioxidant activity, these beet powders could have beneficial effects on consumer health.

## Figures and Tables

**Table 1:** Physicochemical characterization of lyophilized extracts of beet (*Beta vulgaris* sp.) (Mean ± Standard Error).

**Powder**	**WAI**	**WSI**	**T_g_** (**°C**)	**ABTS** (**mM TE/100 g**)	**DPPH** (**mM TE/100 g**)
**B**	44.007 ± 1.000 ^a^	9.051 ± 0.016 ^b^	18.340 ± 0.953 ^c^	0.907 ± 0.001 ^a^	0.447 ± 0.001 ^a^
**M**	27.095 ± 6.096 ^b^	9.374 ± 0.014 ^a^	61.633 ± 1.261 ^a^	0.741 ± 0.001 ^b^	0.295 ± 0.001 ^b^
**I**	30.684 ± 3.427 ^b^	8.955 ± 0.023 ^c^	27.593 ± 0.382 ^b^	0.647 ± 0.001 ^c^	0.208 ± 0.001 ^c^
**Powder**	**TP** (**mg GAE/g**)	**TB** (**mg/100 g**)	**BC** (**mg/100 g**)	**BX** (**mg/100 g**)	**TPC** (**µg/mL**)
**B**	12.354 ± 0.001 ^a^	382.351 ± 0.092 ^a^	219.175 ± 0.092 ^a^	163.176 ± 0.001 ^a^	5.974 ± 0.001 ^a^
**M**	6.093 ± 0.001 ^b^	15.718 ± 0.016 ^b^	10.001 ± 0.001 ^b^	5.717 ± 0.006 ^b^	3.524 ± 0.001 ^c^
**I**	5.975 ± 0.001 ^c^	10.110 ± 0.016 ^c^	6.279 ± 0.001 ^c^	3.831 ± 0.006 ^c^	3.655 ± 0.001 ^b^

B = Unencapsulated beet extract. M = Beet extract encapsulated with Maltodextrin. I = Beet extract encapsulated with Inulin. WAI = Water Absorption Index, WSI = Water Solubility Index, T_g_ = Glass Transition Temperature, Antioxidant Activity by 2,2’-azino-bis(3-ethylbenzothiazoline-6-sulfonic acid)) (ABTS), and 2,2-diphenyl-1-picrylhydrazyl (DPPH), TP = Total Polyphenols, TB = Total Betalains, BC = Betacyanins, BX = Betaxanthins and TPC = Total protein concentration. ^a, b, c^ Different literals between rows indicate significant statistical difference (*p* < 0.05).

**Table 2:** Correlation coefficient and the level of significance (*p*-value) between the response variables of encapsulated freeze-dried beet extracts (*Beta vulgaris* sp.).

	WSI	T_g_	ABTS	DPPH	TP	TB	BC	BX	TPC
**WAI**	−0.4519	−0.7410	0.8968	0.8963	0.8933	0.8882	0.8878	0.8887	0.8982
*p*-value	0.2220	0.0224	0.0011	0.0011	0.0012	0.0014	0.0014	0.0014	0.0010
**WSI**		0.9068	−0.6166	−0.6188	−0.3118	−0.2838	−0.2817	−0.2865	−0.3412
*p*-value		0.0007	0.0770	0.0756	0.4141	0.4593	0.4627	0.4549	0.3689
**T_g_**			−0.8872	−0.8885	−0.6768	−0.6549	−0.6533	−0.6571	−0.6995
*p*-value			0.0014	0.0014	0.0453	0.0556	0.0564	0.0545	0.0360
**ABTS**				1.0000	0.9398	0.9293	0.9285	0.9304	0.9500
*p*-value				<0.0001	0.0002	0.0003	0.0003	0.0003	<0.0001
**DPPH**					0.9388	0.9283	0.9275	0.9293	0.9491
*p*-value					0.0002	0.0003	0.0003	0.0003	<0.0001
**TP**						0.9996	0.9995	0.9997	0.9995
*p*-value						<0.0001	<0.0001	<0.0001	<0.0001
**TB**							1.0000	1.0000	0.9982
*p*-value							<0.0001	<0.0001	<0.0001
**BC**								1.0000	0.9980
*p*-value								<0.0001	<0.0001
**BX**									0.9983
*p*-value									<0.0001

WAI = Water absorption index, WSI = Water solubility index, T_g_ = Glass transition temperature, ABTS and DPPH = Antioxidant activity, TP = Total polyphenols, TB = Total betalains, BC = Betacyanins, BX = Betaxanthins and TPC = Total protein concentration. *p*-value = Level of significance of the correlation.
